# Efficacy of Integrated Yoga and Naturopathy With Physiotherapy or Acupuncture for Low Back Pain: A Parallel, Two-Arm, Randomized Trial

**DOI:** 10.7759/cureus.55198

**Published:** 2024-02-29

**Authors:** Sofia Mudda, Sujatha K Janardhan, Chenchula Santenna, Aruchunan Mooventhan, Prashanth Shetty

**Affiliations:** 1 Yoga and Naturopathy, Ayurveda, Yoga and Naturopathy, Unani, Siddha, Sowa-Rigpa, and Homoeopathy (AYUSH) All India Institute of Medical Sciences, Bhopal, IND; 2 Natural Therapeutics, Sri Dharmasthala Manjunatheshwara (SDM) College of Naturopathy and Yogic Sciences, Mangalore, IND; 3 Pharmacology, All India Institute of Medical Sciences, Bhopal, IND; 4 Research, Government Yoga and Naturopathy Medical College and Hospital, Chennai, IND; 5 Yoga, Sri Dharmasthala Manjunatheshwara (SDM) College of Naturopathy and Yogic Sciences, Mangalore, IND

**Keywords:** state and trait anxiety, range of motion, naturopathy, yoga, physiotherapy, low back pain, acupuncture

## Abstract

Introduction: Low back pain (LBP) is a musculoskeletal condition that affects many individuals. While physiotherapy and acupuncture are frequently used treatments, determining their specific contributions when used alongside integrated yoga and naturopathy (IYN) therapy for relieving chronic LBP symptoms and enhancing quality of life is important.

Methodology: In the present prospective randomized comparative trial, a total of 60 participants were divided into the following two groups: physiotherapy with IYN and acupuncture with IYN. The treatment duration was 10 days, and assessments were conducted both before (baseline) and after the intervention. Various assessment tools, such as the visual analog scale (VAS), Oswestry Disability Index (ODI), fingertip-to-floor test (FTF), State-Trait Anxiety Inventory (STAI), and Short Form 36 (SF-36), were utilized. The statistical analysis was performed using SPSS version 21.0 (Armonk, NY: IBM Corp).

Results: The results revealed significant differences in VAS score, ODI, FTF score, STAI score, and SF-36 score between the physiotherapy and acupuncture groups (p<0.001). A t-test for the equality of means and a Mann-Whitney U test were used to compare the two groups; these tests revealed a significant difference in disability levels, range of motion, and state of anxiety. The acupuncture group showed a significant difference in the ODI (15.9 {12.6, 19.3}; p <0.001) and state anxiety (23.0 {20.0, 26.0}; p<0.001) compared to the physiotherapy ODI (22.4 {18.5, 26.4}) and state anxiety (27.5 {25.0, 30.0}). The physiotherapy group showed a significant difference in range of motion (7.7 {5.7, 9.7}; p<0.001) compared to the acupuncture group (11.6 {9.8, 13.5}).

Conclusion: The present study findings revealed that both physiotherapy and acupuncture, as interventions along with integrated yoga and naturopathy may be considered an effective treatment strategy for chronic LBP.

## Introduction

Low back pain (LBP) is one of the routinely reported reasons for work absenteeism and has become a significant welfare and economic problem due to the disability associated with chronic back pain [1]. LBP was defined as pain, a tense muscle, or stiffness confined below the costal margin and above the inferior gluteal folds. The cause may also include the involvement of the sciatic nerve. Chronic LBP (CLBP) can persist for more than 12 weeks and is the leading cause of years lived with disability (YLD) on a global scale [2]. In 2020, there were more than 500 million cases of low back pain globally, constituting a substantial 7.7% of all YLDs and thus the greatest effect on the burden of disability worldwide. It is expected that globally, more than 800 million people will suffer from back pain by 2050. This surge is anticipated to be particularly pronounced across regions of Asia and Africa [3]. LBP is the fifth most common reason for physician visits and the second most common symptomatic reason after upper respiratory symptoms in the United States [4]. The lifetime incidence of LBP is 66% (56-75%), with a point prevalence of 48% (40-56%), the highest among females, the rural population, and elementary workers in India, and an annual prevalence of 51% (45-58%) [5]. Many environmental and personal factors can have an impact on the onset and course of low back pain [6]. Furthermore, frequently mentioned risk factors include stress, anxiety, depression, job dissatisfaction, low levels of social support at work, and whole-body vibration exposure [7].

Recent clinical guidelines have introduced significant updates and underscored recommendations that continue to be endorsed in managing nonspecific LBP. Key messages from these guidelines include the discouragement of routine imaging, a focus on initial simple care with prompt follow-up, and a preference for nonpharmacological interventions over pharmacological interventions [8]. There is an extensive range of treatment opportunities for patients with LBP, including medication, physical modalities, manual therapy, exercise, lifestyle, and behavioral therapies [9]. The duration of LBP is a major factor that must be considered when choosing a suitable therapeutic modality [10]. Complementary and alternative medicine (CAM) practices are becoming more widespread as they are perceived as safer and more holistic methods for managing various features of musculoskeletal disorders. Chronic pain is the primary reason for seeking out these alternative medical therapies. CAM serves as a supportive and unconventional approach that effectively mitigates discomfort and disability linked predominantly to chronic conditions, enabling individuals to better manage their day-to-day activities [11-13]. Currently, CAM is being utilized for a range of musculoskeletal conditions, such as low back ache, neck pain, osteoarthritis, and fibromyalgia [14].

Yoga and naturopathy are officially recognized in India as indigenous systems of medicine by the Government of India's Ministry of Ayurveda, Yoga and Naturopathy, Unani, Siddha, Sowa-Rigpa, and Homoeopathy (AYUSH) [15]. In a typical inpatient facility for yoga and naturopathy, various therapies, including massage therapy, hydrotherapy, diet therapy, heliotherapy, acupressure, acupuncture, and yoga therapy, are provided [16]. Integrated yoga and naturopathy (IYN) is a comprehensive therapeutic approach that combines the principles and practices of yoga and naturopathy to address various health conditions, including low back pain [17,18]. Studies investigating treatment modalities used in integrative yoga and naturopathy (IYN), including massage, hydrotherapy, dietary interventions, acupuncture, and yoga, have demonstrated favorable outcomes in mitigating symptoms related to low back pain [19-24].

The findings of a randomized trial on naturopathic care for chronic low back pain (CLBP) by Szczurko et al. showed that naturopathic care provides considerably better progress than physiotherapy advice among patients with chronic LBP [25]. Both physiotherapy and acupuncture are commonly used either as standalone treatments or in combination with yoga and naturopathy for low back pain [25]. Previous research has shown that naturopathic care is more profitable than a standardized physiotherapy education regimen for chronic LBP [26]. A systematic review by Cramer et al. yielded robust evidence supporting the short-term efficacy of and moderate evidence supporting the long-term efficacy of yoga in patients with chronic low back pain, with a focus on patient-centered outcomes [24]. Individual studies conducted on acupuncture and physiotherapy for low back pain suggest significant improvement [10,27].

The management of LBP varies among individuals due to divergent responses to treatment approaches, and a single intervention is seldom completely effective [28]. In the case of CLBP, an optimal strategy involves a multidisciplinary and systematic approach encompassing a range of medical, psychological, physical, and interventional modalities to maximize therapeutic efficacy [28]. A systematic review assessing the outcomes of multimodal treatments administered by North American naturopathic physicians suggested that although several studies had limited sample sizes, the results consistently indicated that the utilization of whole-system naturopathic medicine was linked to enhanced health outcomes and improved quality of life among patients with chronic conditions or those at risk of developing them [29]. According to Arankalle's review, a wide range of people with musculoskeletal disorders may benefit greatly from implementing a naturopathy and yoga protocol that includes lifestyle changes, therapeutic fasting, dietary modifications, and modalities, such as hydrotherapy, mud therapy, massage, physiotherapy, yoga, and exercise therapy [18]. This strategy is consistent with the results of a systematic scoping review that showed that whole-system, multimodality naturopathic medicine works well in treating a variety of illnesses, including type 2 diabetes, depression, anxiety, polycystic ovary syndrome, cardiovascular disease, musculoskeletal pain, and other complex chronic ailments [30].

The use of a comprehensive approach in yoga and naturopathy often includes acupuncture and physiotherapy and is common in India. However, the literature on acupuncture and physiotherapy focuses on these treatments in isolation rather than in combination with yoga and naturopathy. Therefore, in this study, we focused on evaluating and comparing the influence of physiotherapy or acupuncture in combination with IYN treatments on reducing LBP symptoms and enhancing quality of life. By investigating the effectiveness of these combined interventions, we seek to provide valuable insights into the optimal management of chronic LBP.

## Materials and methods

Study design

This is a prospective randomized comparative trial to evaluate the efficacy of integrated yoga and naturopathy when used as an adjuvant to physiotherapy or acupuncture in the management of chronic LBP. There were equal numbers of subjects (30 each) in group 1 (physiotherapy with IYN) and group 2 (acupuncture with IYN). The data were measured at baseline and after 10 days. The study duration was 12 months.

Study settings

The study population was chosen from the inpatient facility of Sri Dharmasthala Manjunatheshwara Yoga and Nature Cure Hospital, Shanthivana, Dharmasthala, following the approval of the Institutional Ethical Committee (approval number: EC-564) at Sri Dharmasthala Manjunatheshwara College of Naturopathy and Yogic Sciences, where the research took place.

Participants

The present study involved a cohort of 60 adults aged between 18 and 55 years who met the diagnostic criteria for low back pain localized between the 12th rib and gluteus fold [2]. These individuals were selected from a pool of 93 adult patients. Chronic low back pain (CLBP) was defined as pain persisting for more than 12 weeks, and only patients with chronic nonspecific low back pain were included [2]. The exclusion criteria included the presence of significant pathology, such as bone fractures, nerve damage, or severe psychiatric conditions, and the presence of clinically diagnosed depression by a physician. Additionally, cases of back pain stemming from other causes associated with systemic disease and neurological disorders were ruled out through diagnostic triage based on medical history and clinical examination. This triage is a crucial guideline in LBP management, aiming to exclude nonspinal causes and categorize patients according to specific spinal pathology (<1%), presence of radicular syndrome (~5-10%), or presence of nonspecific LBP (NSLBP; 90-95%), with NSLBP diagnosed by exclusion of the first two categories [8,31].

**Figure 1:**
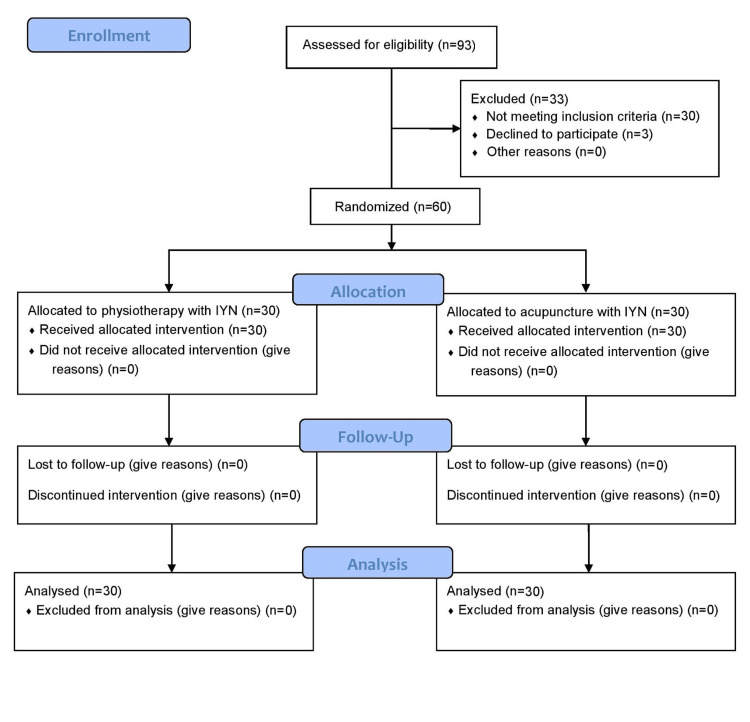
The Consolidated Standards of Reporting Trials (CONSORT) flow diagram.

Additionally, individuals with a history of direct impact trauma, inflammatory arthritis of large joints, pregnancy, recent exposure to yoga and naturopathy, physiotherapy or acupuncture within the past six months, and obesity related to back pain were excluded because earlier exposure to these therapies initiates their effects on alleviating the symptoms of LBP and may interfere with the outcome variables of the study. The potential participants were provided with both verbal and written details regarding the study. Those who chose to participate formally endorsed the informed consent documents. The baseline characteristics of the participants were measured, as can be seen in Table 1. The Consolidated Standards of Reporting Trials (CONSORT) flow diagram trial progress is illustrated in Figure 1.

**Table 1:** Baseline characteristics of the study participants (n=60). NCD: noncommunicable disease

Variable	Frequency	Percentage
Gender of the participant		
Male	16	26.7
Female	44	73.3
Age group		
18-40 year	14	23.3
41-55 year	46	76.7
Socioeconomic status		
Lower	46	76.7
Middle	14	23.3
Upper	0	-
Demographic location		
Rural	17	28.3
Urban	43	71.7
NCD status		
Nil	44	47.8
Cardiovascular disease	14	15.2
Gastrointestinal disorders	7	7.6
Endocrine and metabolic disorders	16	17.4
Reproductive tract issues	2	2.2
Respiratory diseases	5	5.4
Others	4	4.3

Intervention

The participants included in the study were randomly allocated to group 1 (physiotherapy with IYN) or group 2 (acupuncture with IYN). Selected yoga and naturopathic treatments specially considered for chronic LBP were given to both test groups. Both groups received two sessions (morning and afternoon) of naturopathy treatments for a duration of 20-75 minutes/day and one session of yoga therapy for a duration of 60 minutes for 10 days. Trained treatment attendants who did not participate in assessing the effects of the treatments applied during hydrotherapy and massage were included. A qualified yoga expert conducted the yoga sessions. Both groups in the study adhered to a specific dietary regimen tailored to individual participant requirements, comprising two days of juice fasting, three days of juice and fruit fasting, two days of raw vegetable diet, and three days of a standard boiled diet.

Group 1

The physiotherapy interventions used for group 1 included interferential therapy, heat or ice therapy, ultrasound, and various core strengthening exercises, such as lowering the stomach to the spine, leg movements, abdominal controlled curls, bridging, wall squats, and single-leg bridging [32-36]. The total duration of the session was 40-45 minutes for 10 days.

Group 2

The acupuncture group received a single session of acupuncture based on traditional Chinese medicine, lasting 15-30 minutes for a period of 10 days. The specific acupuncture points utilized were Du 20, LI 4, UB 11, Du3, Du 4, UB 40, UB 60, and St 44, sourced from different meridians in addition to Ah-shi points targeting low back pain. The aim was to achieve the sensation of "de qi," as outlined in traditional texts and previous studies [37,38]. This acupuncture procedure was administered by two naturopathy physicians with institutional qualifications and three years of experience in therapeutic acupuncture. The participants’ involvement was limited to the administration of acupuncture, and they had no other role in the trial. Bilateral manual needling was performed without any additional stimulation. Puncturing was carried out using one locally manufactured cun filiform needle made of stainless steel; the needle was 0.38 mm in diameter and 25 mm in length. The tables in the appendix present the protocol which was specially planned for chronic LBP.

Assessments

In the present study, data were collected using three primary outcome variables and two secondary outcome variables. The primary objectives of the present study included severity of pain, disability level of back pain, and range of motion. The secondary objectives were to assess state-trait anxiety and quality of life. The assessment was performed using rating scales such as the visual analog scale (VAS) for the severity of pain, the Oswestry Disability Index (ODI) for disability index, the fingertip-to-floor test (FTF) for range of motion, and the State-Trait Anxiety Inventory (STAI) scale for state and trait anxiety and the Short Form 36 (SF-36) scale for assessing health-related quality of life (HRQoL).

Individuals with chronic pain face a high risk of concurrent development of anxiety and depression, a susceptibility that is notably more pronounced in females [39]. Given the intricate relationship between anxiety and chronic pain, it is reasonable to incorporate comprehensive screening for anxiety symptoms as an integral component of initial patient assessment. This proactive approach is essential not only for optimizing treatment but also for holistic management of the patient, ultimately contributing to a more favorable prognosis [40]. Furthermore, chronic LBP exerts a substantial impact on the well-being of workers. This impact manifests in various forms, including depression, anxiety, job dissatisfaction, and a pervasive fear of employment termination, all of which culminate in a compromised quality of life [41]. Hence, we used the State-Trait Anxiety Inventory scale (STAI) to assess current state and trait anxiety levels and the Short Form 36 scale to assess health-related quality of life as secondary outcome variables.

Primary outcome variables

Visual Analog Scale

The visual analog scale (VAS) offers a constant scale for subjective magnitude assessment, employing a linear configuration wherein verbal descriptors demarcate the endpoints of the specific symptom under evaluation [42]. The line is usually 10 centimeters long and horizontal [43], and variations in length and orientation have been explored and validated as effective alternatives [44,45]. In this study, participants were asked to rate the severity of pain associated with low back pain via a visual assessment technique on a scale ranging from 0 to 10 cm.

The Oswestry Disability Index

The Oswestry Disability Index (ODI) is considered the gold standard for evaluating the severity of back pain. The questionnaire comprises 10 items and is designed to discern the extent to which back or leg pain impairs a patient's ability to manage their daily activities [46].

Fingertip-to-Floor Test

The fingertip-to-floor (FTF) test serves as a tool for evaluating total mobility during forward flexion in a standing position. This assessment entails the measurement of the vertical distance between the tip of the middle finger and the floor, quantified in centimeters through the use of a standardized measuring tape. These measurements were performed three times, and the mean values were recorded. Notably, this test demonstrated excellent validity, reliability, and responsiveness [47].

Secondary outcome variables

State-Trait Anxiety Inventory

The State-Trait Anxiety Inventory (STAI) constitutes a duly validated self-report assessment device comprising 40 items that distinctly encapsulate evaluations of both transient state anxiety and enduring trait anxiety. This inventory has consistently exhibited commendable metrics of validity, internal consistency, and reliability and has been extensively used in research in diverse disciplines, including psychology and medicine [48,49].

Short Form 36

The Short Form 36 (SF 36) is a concise yet versatile health survey with only 36 questions. Its outcome comprises an eight-point scale that delineates profiles of functional health and well-being scores. Additionally, it furnishes psychometrically derived summative evaluations of physical and mental health, alongside a health utility index grounded in individual preferences [50,51].

Baseline data were obtained on the first day, and post-intervention were collected at the end of 10 days. There were no known side effects or adverse events in either group. Severe, unexpected, and possibly related adverse events were monitored and recorded if any occurred in the case records of the participants.

Sample size

This was a parallel, two-arm, prospective, randomized trial wherein the treatment distribution was unblinded but the outcome assessment was blinded. In accordance with suggestions to detect a minimum clinically significant difference of six points in the main outcome measure (Oswestry Disability Index) and aiming for a power of 0.8 with an alpha of 0.05, each group would require 28 participants [52,53]. Factoring a 10% dropout rate, it was determined that a total of 30 patients should be initially recruited into each group. A total of 60 participants with LBP were enrolled in this study, 30 of whom were assigned to each group.

Randomization and blinding

To ensure unbiased allocation, an automated, web-based randomization service (www.random.org) was used to generate the randomization list. Allocation concealment was performed using a sequentially numbered opaque sealed envelope. Following the baseline assessment, eligible participants were sequentially assigned by the investigator to group 1 (physiotherapy with IYN) or group 2 (acupuncture with IYN) at a 1:1 ratio. Due to the active nature of the interventions, physiotherapy, and acupuncture, blinding of group assignments was not feasible. However, the personnel responsible for outcome assessment and the statistician analyzing the data were unaware of the group assignment.

Data analysis

The collected data were systematized using Microsoft Excel Sheets (version 2010). The data were analyzed using SPSS version 21.0 software (Armonk, NY: IBM Corp.). The normality of the data was assessed with the Kolmogorov-Smirnov test. For data that were not normally distributed, the Mann-Whitney test was used, while normally distributed data were analyzed via a different analytical approach. Within-group differences for both physiotherapy with integrated yoga and naturopathy and acupuncture with integrated yoga and naturopathy were assessed using paired samples t-tests and Wilcoxon signed-rank tests, depending on the nature of the data. The overall comparison between the two groups was carried out using t-tests for the equality of means and the Mann-Whitney U test. All analyses are presented with 95% confidence intervals (CIs), and statistical significance was set at p≤0.05.

## Results

A total of 93 participants were screened, 60 of whom met the inclusion and exclusion criteria of the study. These 60 patients composed the study sample (n=60). A total of 73.3% (n=44) of the study participants were females, and most were aged 41-55 years (76.7%, n=46). The means±standard deviations of age in physiotherapy with integrated yoga and naturopathy and acupuncture with integrated yoga and naturopathy were 46.3±8.4 and 45.1±10.2, respectively, with variances of 70.7 and 103.4, respectively. The difference between the two groups was not statistically significant according to the unpaired t-test (t=0.48, p=0.6). Thus, the two groups were not significantly different from one another in terms of mean age. The use of yoga and naturopathy interventions across sociodemographic and health status categories was noted among the study participants (Table 1).

This study aimed to assess the short-term impact of combining physiotherapy and acupuncture with integrated yoga and naturopathy (IYN) on pain severity, disability index, range of motion, momentary anxiety level, and quality of life in patients with low back pain. The results were evaluated by comparing data within two following groups: group 1 (physiotherapy with IYN) and group 2 (acupuncture with IYN), with information collected both at baseline and after the intervention (Tables 2, 3).

**Table 2:** Represents changes in visual analog scale (VAS), Oswestry Disability Index (ODI), and fingertip-to-floor test (FTF) of group 1 and group 2. ^a^Median (interquartile range). ^b^P-value for within-group comparisons obtained by Wilcoxon signed-rank test. ^c^P-value for within-change/between-group differences obtained by sample t-test. ^d^P-value for between group change obtained from Mann-Whitney U test. NA denotes statistical test was not applicable; data expressed as estimated mean (95% confidence interval {CI}); group 1: physiotherapy with integrated yoga and naturopathy (IYN), n=30; group 2: acupuncture with integrated yoga and naturopathy (IYN), n=30. NCD: noncommunicable disease

Variables	Assessment	Group 1: physiotherapy with IYN within-group analysis (n=30)	Group 2: acupuncture with IYN within-group analysis (n=30)	Effect size (between group d)	p-Value		
					Within the group analysis		Between the group analysis
					Group 1: physiotherapy with IYN	Group 2: acupuncture with IYN	
Visual analog scale (VAS)^a^	Before	5.5 (5.0, 7.1)	5.0 (5.0, 6.1)	NA	<0.001^b^	<0.001^b^	0.982^d^
	After	1.9 (1.4, 2.6)	2.2 (1.3, 2.5)				
Oswestry Disability Index (ODI)	Before	36.4 (31.9, 40.8)	24.1 (20.8,27.5)	0.7	<0.001^c^	<0.001^c^	0.013^c^
	After	22.4 (18.5, 26.4)	15.9 (12.6, 19.3)				
Fingertip-to-floor test (FTF)	Before	14.4 (11.4, 17.3)	18.6 (16.3, 20.8)	0.8	<0.001^c^	<0.001^c^	0.004^c^
	After	7.7 (5.7, 9.7)	11.6 (9.8, 13.5)				

**Table 3:** Represents changes in State-Trait Anxiety Inventory (STAI) and Short Form 36 (SF-36) of group 1 and group 2. ^a^Median (interquartile range). ^b^P-value for within-group comparisons obtained by Wilcoxon signed-rank test.​​​​​​ ^c^P-value for within-change/between-group differences obtained by sample t-test. ^d^P-value for between group change obtained from Mann-Whitney U test. Data expressed as estimated mean (95% confidence interval {CI}); group 1: physiotherapy with integrated yoga and naturopathy (IYN), n=30; group 2: acupuncture with integrated yoga and naturopathy (IYN), n=30; NA denotes statistical test was not applicable

Variables		Assessment	Group 1: physiotherapy with IYN within-group analysis (n=30)	Group 2: acupuncture with IYN within-group analysis (n=30)	Effect size (between group d)	p-Value		
						Within the group analysis		Between the group analysis
						Group 1: physiotherapy with IYN	Group 2: acupuncture with IYN	
State-Trait Anxiety Inventory (STAI)	State anxiety^a^	Before	35.0 (31.3, 45.0)	30.0 (27.0, 34.0)	NA	<0.001^b^	<0.001^b^	0.001^d^
		After	27.5 (25.0, 30.0)	23.0 (20.0, 26.0)				
	Trait anxiety	Before	38.6 (35.2, 41.9)	35.0 (32.0, 37.9)	0.1	<0.001^c^	0.002^c^	0.694^c^
		After	31.2 (28.9, 33.6)	30.6 (28.2, 32.9)				
SF-36								
Physical functioning (PF)^a^		Before	75.0 (70.0, 85.0)	80.0 (75.0, 85.0)	NA	<0.001^b^	<0.001^b^	0.245^d^
		After	85.0 (75.0, 93.8)	90.0 (80.0, 95.0)				
Role physical (RP)^a^		Before	50.0 (0.0, 75.0)	50.0 (25.0, 75.0)	NA	0.001^b^	<0.001^b^	0.170^d^
		After	87.5 (50.0, 100.0)	100.0 (75.0, 100.0)				
Role emotional (RE)^a^		Before	66.7 (8.3, 100.0)	100.0 (66.7, 100.0)	NA	0.001^b^	0.016^b^	0.695^d^
		After	100.0 (100.0, 100.0)	100.0 (100.0, 100.0)				
Vitality (VT)		Before	60.8 (55.6, 66.1)	67.2 (63.3, 71.2)	0.1	<0.001^c^	<0.001^c^	0.825^c^
		After	78.5 (74.4, 82.6)	77.9 (74.7, 81.1)				
Mental health (MH)^a^		Before	76.0 (56.0, 87.0)	80.0 (68.0, 92.0)	NA	<0.001^b^	<0.001^b^	0.261^d^
		After	90.0 (81.0, 92.0)	92.0 (88.0, 92.0)				
Social functioning (SF)^a^		Before	87.5 (62.5, 100.0)	75.0 (62.5, 100.0)	NA	0.004^b^	0.002^b^	0.233^d^
		After	100.0 (75.0, 100.0)	87.5 (75.0, 100.0)				
Bodily pain (BP)^a^		Before	45.0 (35.0, 57.5)	45.0 (45.0, 57.5)	NA	<0.001^b^	<0.001^b^	0.388^d^
		After	73.8 (67.5, 77.5)	67.5 (65.0, 90.0)				
General health (GH)^a^		Before	70.0 (51.3, 75.0)	70.0 (65.0, 80.0)	NA	0.001^b^	<0.001^b^	0.201^d^
		After	77.5 (66.3, 88.8)	80.0 (75.0, 90.0)				

There was a significant difference between the results of physiotherapy with IYN group and acupuncture with IYN group at the end of 10 days after the treatment in the scores of visual analog scale (VAS) (median {IQR}; 1.9 {1.4, 2.6} and 2.2 {1.3,2.5}), Oswestry Disability Index (ODI) (estimated mean {95% CI}; 22.4 {18.5, 26.4} and 15.9 {12.6, 19.3}), and fingertip-to-floor test (FTF) (estimated mean {95% CI}; 7.7 {5.7, 9.7} and 11.6 {9.8,13.5}). There was a significant difference between group 1 and group 2 in terms of secondary objectives such as state and trait anxiety and quality of life (p<0.001) (Table 3).

**Figure 2:**
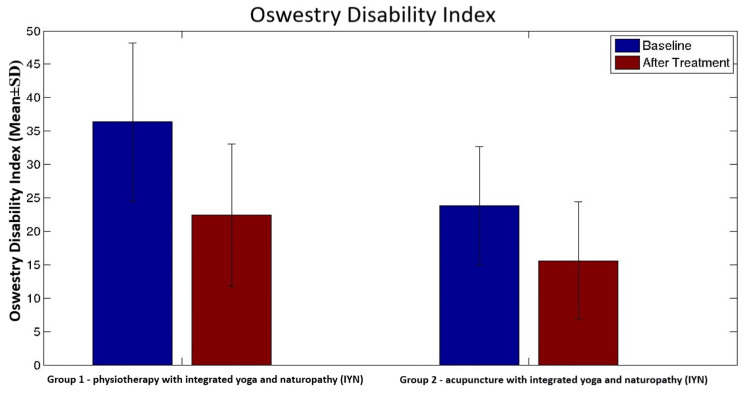
Bar graph showing comparison of means of Oswestry Disability Index scores of both groups with error bars showing standard deviation. The Y-axis shows a mean reduction in disability scores. The means of group 1 and group 2 are compared using a bar graph with error bars showing standard deviations. The p-value within the group analysis is <0.001.

**Figure 3:**
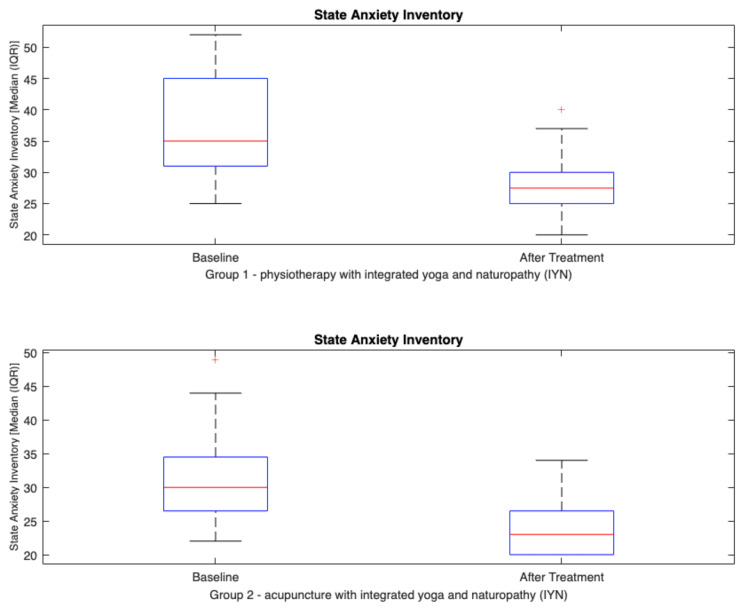
Box plot showing state anxiety scores of group 1 and group 2. The Y-axis shows a reduction in state (at the moment) anxiety scores. The p-value within the group analysis is <0.001.

Overall comparisons between groups were performed by using a t-test for equality of means and the Mann-Whitney U test, which showed significant differences in the Oswestry Disability Index, range of motion, and state anxiety. The acupuncture with IYN group showed a significant difference in ODI (estimated mean {95% CI}: 15.9 {12.6, 19.3}; p<0.001) and state anxiety (median {IQR}: 23.0 {20.0, 26.0}; p<0.001) compared to the physiotherapy with IYN group ODI (estimated mean {95% CI}: 22.4 {18.5, 26.4}) and state anxiety (median {IQR}: 27.5 {25.0, 30.0}) (Figures 2, 3).

**Figure 4:**
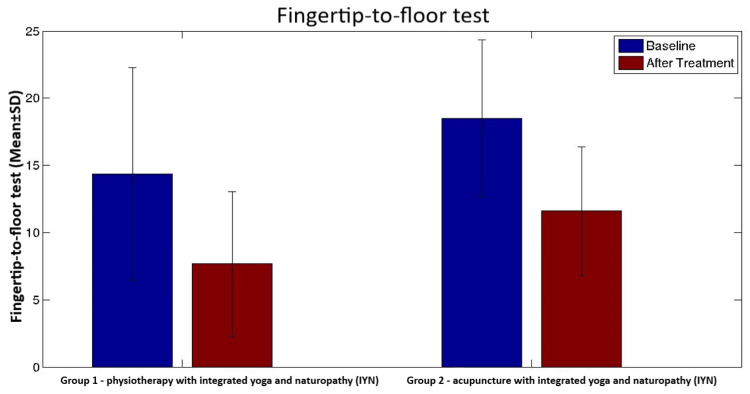
Bar graph showing comparison of means of fingertip-to-floor test scores of both groups with error bars showing standard deviation. The Y-axis shows a mean reduction in fingertip-to-floor test scores. The means of group 1 and group 2 are compared using a bar graph with error bars showing standard deviations. The p-value within the group analysis is <0.001.

However, there was a significant difference in the range of motion in physiotherapy with IYN group (estimated mean {95% CI}: 7.7 {5.7, 9.7}; p<0.001) when compared to the acupuncture with IYN group (estimated mean {95% CI}: 11.6 {9.8, 13.5}) (Figure 4).

## Discussion

To the best of our knowledge, this is the first study comparing the effects of physiotherapy or acupuncture in combination with Integrated yoga and naturopathy in the management of chronic LBP. The present study findings revealed a significant effect within the groups in terms of pain severity, disability index, range of motion, state and trait anxiety rates, and quality of life (Tables 2, 3). This could be largely attributed to the comprehensive treatment protocol of integrated yoga and naturopathy (IYN), as neither of the groups was significantly different from the other in terms of mean age or in terms of patient adherence to the protocol.

In the present study, discerning the specific impact of an individual treatment modality responsible for the observed enhancement in outcome measures within both groups proved challenging. However, it is commonly acknowledged that a single intervention does not typically provide comprehensive effectiveness for all individuals suffering from chronic low back pain. As a result, a multidisciplinary approach to treatment is often recommended [11,28]. The chosen treatment protocol, which integrates yoga and naturopathy, has the potential to provide significant benefits to a substantial portion of CLBP patients. This is because both yoga and naturopathy modalities have been shown to have substantial efficacy in treating CLBP patients. Yoga, as a form of mind-body therapy, can enhance body flexibility, endurance, and good control of the mind over the body [11]. Thus, yoga yields significant and lasting reductions in low back pain accompanied by improvements in disability [24], functional capabilities, and psychosocial well-being over both short-term and long-term periods [54]. Furthermore, naturopathy is a well-accepted and safe modality [11]. The results of a randomized trial investigating naturopathic care for CLBP demonstrated that naturopathic care significantly improved disability levels, HRQoL, range of motion (ROM), weight loss, and body mass index when compared to physiotherapy advice among individuals suffering from chronic LBP [25].

The efficiency of naturopathic modalities is substantiated by the biological plausibility of these methods reported in previous literature [19,20,55-60]. For example, the mechanisms by which a wide range of diseases respond to hydrotherapy can likely be categorized into mechanical, thermal, and chemical effects [19,20,55]. Hydrotherapy has a traditional role in stimulating processes such as digestion, circulation, and the immune system [20]. Additionally, it is employed for pain relief, stress reduction, and rejuvenation of the body [56,57]. Massage therapy is a widely employed therapeutic practice for addressing a range of acute and chronic clinical conditions, such as shoulder pain, sprains, knee osteoarthritis, and backache. The proposed mechanisms of action underlying massage therapy include an increase in lactate clearance, an increase in lymphatic flow, modulation of the immune system, and cognitive function; prevention of fibrosis; and a reduction in anxiety and depression [58-60].

In India, thus, yoga in combination with naturopathy is a common practice followed at various yoga and naturopathy hospitals to manage various diseases [16]. A retrospective study conducted by Maheshkumar et al. focused on investigating trends in the utilization of yoga and naturopathy-centered lifestyle clinics for the management of NCDs in the South Indian state of Tamil Nadu, which revealed that Lumbago was the second most prevalent individual medical condition among patients seeking consultations at medical college hospital facilities (80%) [61]. Furthermore, patients may have co-opted to undergo these therapies because of their comorbid conditions, as an integrated approach is reported to reduce the burden of NCDs [17]. Therefore, in the present study, the effectiveness of a comprehensive protocol of integrated yoga and naturopathy for alleviating the symptoms of CLBP was evaluated.

The current study revealed significant differences in between-group effectiveness in terms of disability level, range of motion (flexibility of the spine), and state of anxiety. Consistent with the results of previous studies, our study also revealed significant improvement in the range of motion in CLBP patients who underwent physiotherapy (Table 2) [62,63]. The underlying mechanism responsible for the observed therapeutic effects of physiotherapy may be attributed to lumbar stabilization exercises designed to enhance neuromuscular control, muscular strength, and endurance of the core muscles crucial for maintaining dynamic spinal and trunk stability [64]. In accordance with the standard physiotherapy regimen for nonspecific LBP, interventions such as hot moist pack and interferential therapy were administered. The application of treatments such as the hot moist pack is aimed at alleviating pain, reducing superficial muscle spasms, and enhancing tissue extensibility. Concurrently, interferential therapy was employed to mitigate pain by acting on the pain gate theory and facilitating neurotransmitter depletion [62].

Our study findings contrast with those of a prospective randomized trial in which improvements in range of motion were found in the acupuncture group compared to the physiotherapy group [65]. However, we found that the range of motion was greater in the physiotherapy group than in the acupuncture group.

In this study, acupuncture was found to be more effective than physiotherapy at improving the disability level of CLBP patients (Table 2). These findings align with those of the study reported by Haake et al., which showed significant improvement in the severity of back-specific disability in CLBP patients in the acupuncture group compared to the physiotherapy group [66]. This finding is substantiated by the mechanism of local effects via intramuscular stimulation and another mechanism involving segmental analgesia [67].

Similarly, in the present study, acupuncture was more effective than physiotherapy at improving the anxiety of CLBP patients (Table 3). These findings align with the study reported by Leibing et al., which showed significant improvement in the psychological distress of CLBP patients in the acupuncture group compared to the physiotherapy group [68]. This finding is supported by the large nonspecific effects of acupuncture that might be underpinned by psychosocial processes [69].

The present study findings showed that using physiotherapy or acupuncture in combination with IYN for a short duration of 10 days is effective in treating CLBP. Although both treatments are effective, physiotherapy is more effective at improving the range of motion, specifically the flexibility of the lumbar spine, while acupuncture has shown improvements in reducing disability severity and psychological distress.

This study has certain limitations, including the absence of a control or standard treatment group, the absence of an adequate sample size, which makes it difficult to generalize the data and limits the applicability of our findings; the lack of screening tools employed in the assessment of individuals with chronic low back pain; the use of subjective variables prone to bias can introduce measurement errors; and the use of a short term follow-up period may limit the ability to observe the long term benefits of treatment. Thus, a randomized controlled trial involving a larger sample size is needed to substantiate these results.

## Conclusions

The findings suggest that both physiotherapy and acupuncture, when combined with integrated yoga and naturopathy, can be effective interventions for individuals experiencing chronic low back pain. Moreover, physiotherapy can be considered for patients with chronic low back pain due to lumbar segmental instability and acupuncture can be considered for chronic low back pain with higher levels of functional disability. The insights gained from this study will help in designing future pragmatic trials in yoga and naturopathy, with the aim of enhancing the reported benefits over extended follow-up periods. Additionally, policymakers should give careful consideration to the potential risks, benefits, challenges, and opportunities associated with the provision of yoga and naturopathic care within the community.

## Appendices

**Table 4:** Naturopathy interventions chosen for chronic low back pain.

Day	Morning therapy (9:30 AM-10:30 AM)	Afternoon therapy (3:00 PM-4:00 PM)
1	Enema (10 min), steam bath (10 min)	Neutral immersion bath with Epsom salt (15 min)
2	Enema (10 min), full body massage (45 min)	Alternate hip bath (20 min)
3	Enema (10 min), sauna bath (10 min)	Neutral spinal bath (15 min)
4	Enema (10 min), neutral douche to whole body (15 min)	Neutral hip bath (15 min)
5	Enema (10 min), Deluxe Hydro massage (20 min)	Neutral half bath with Epsom salt (15 min)
6	Enema (10 min), partial massage to back, abdomen, and legs with infrared light to lower back (30 min)	Revulsive compress to lower back (10 min)
7	Neutral underwater massage (15 min)	Neutral spinal spray (15 min)
8	Reclining steam bath (10 min)	Kidney pack (20 min)
9	Full mud bath (20 min)	Neutral hip bath (15 min)
10	Cold circular jet (10 min)	Revulsive compress to lower back (10 min)

**Table 5:** Diet protocol planned for chronic low back pain.

Day	7:30 AM	9:00 AM	11:00 AM	2:00 PM	4:00 PM	6:30 PM
1	Lemon honey juice (200 mL)	Finger millet porridge (100 g)	Boiled unpolished rice (150 g), boiled vegetables (200 g), papaya (100 g), buttermilk (100 mL)	Lemon honey juice (200 mL)	Vegetable salad (200 g), sprouts (25 g)	Vegetable soup (200 mL), raw vegetables (200 g), sprouts (25 g)
2	Lemon honey juice (200 mL)	Ash gourd juice (200 mL)	Lemon honey juice (200 mL)	Ash gourd juice (200 mL)	Lemon honey juice (200 mL)	Ash gourd juice (200 mL)
3	Lemon honey juice (200 mL)	Ash gourd juice (200 mL)	Carrot Juice (200 mL)	Lemon honey juice (200 mL)	Ash gourd juice (200 mL)	Sweet lime juice (200 mL)
4	Lemon honey juice (200 mL)	Carrot juice (200 mL)	Papaya (200 g), buttermilk (200 mL)	Lemon honey juice (200 mL)	Carrot juice (200 mL)	Apple (200 g), vegetable soup (200 mL)
5	Lemon honey juice (200 mL)	Carrot juice (200 mL)	Papaya (200 g), buttermilk (200 mL)	Lemon honey juice (200 mL)	Carrot juice (200 mL)	Apple (200 g), vegetable soup (200 mL)
6	Lemon honey juice (200 mL)	Carrot juice (200 mL)	Papaya (200 g), buttermilk (200 mL)	Lemon honey juice (200 mL)	Carrot juice (200 mL)	Apple (200 g), vegetable soup (200 mL)
7	Lemon honey juice (200 mL)	Carrot juice (200 mL)	Raw vegetables (200 g), sprouts (25 g), buttermilk (200 mL)	Lemon honey juice (200 mL)	Carrot juice (200 mL)	Vegetable soup (200 mL), raw vegetables (200 g), sprouts (25 g)
8	Lemon honey juice (200 mL)	Carrot juice (200 mL)	Raw vegetables (200 g), sprouts (25 g), buttermilk (200 mL)	Lemon honey juice (200 mL)	Carrot juice (200 mL)	Vegetable soup (200 mL), raw vegetables (200 g), sprouts (25 g)
9	Lemon honey juice (200 mL)	Finger millet porridge (100 g)	Boiled unpolished rice (150 g), boiled vegetables (200 g), papaya (100 g), buttermilk (200 mL)	Carrot juice (200 mL)	Lemon honey juice (200 mL)	Vegetable soup (200 mL), whole wheat Indian flat bread-2 (25 g), boiled vegetables (200 g), apple (200 g)
10	Lemon honey juice (200 mL)	Finger millet porridge (100 g)	Boiled unpolished rice (150 g), boiled vegetables (200 g), papaya (100 g), buttermilk (200 mL)	Carrot juice (200 mL)	Lemon honey juice (200 mL)	Vegetable soup (200 mL), whole wheat Indian flat bread-2 (25 g), boiled vegetables (200 g), apple (200 g)

**Table 6:** Structured yoga module for chronic low back pain.

S. no.	Yogasanas	Duration
1	Loosening exercises	10 min
1.1	Head and neck movements, shoulder rotation, and elbows flexion and extension	
1.2	Wrist up and down, rotation movements, finger clenching	
1.3	Spinal twist, hip up and down, lateral bending, rotational movements	
1.4	Leg forward swing, sideward swing, and knees flexion and extension	
1.5	Ankle up and down, rotation, toes clenching	
2.	Breathing exercises	6 min
2.1	Hands in and out breathing exercise and hands stretch breathing exercise	
2.2	Ankle stretch breathing exercise and tiger breathing exercise	
3	Standing series	6 min
3.1	Tadasana	
3.2	Katichakrasana	
3.3	Ardhachakrasana	
4	Supine series	8 min
4.1	Folded legs lumbar stretch	
4.2	Crossed leg lumbar stretch	
4.3	Pawanamuktasana	
4.4	Setubandhasana with breathing	
5	Sitting series	6 min
5.1	Vakrasana	
5.2	Vajrasana	
5.3	Ushtrasana	
6	Prone series	6 min
6.1	Bhujangasana	
6.2	Dhanurasana	
6.3	Makarasana	
7	Pranayama	10 min
7.1	Nadishodhana pranayama	
7.2	Bhramari pranayama	
8	Relaxation technique	8 min
8.1	Deep relaxation technique (DRT)	
Total duration		60 min

**Table 7:** Physiotherapy interventions selected for chronic low back pain.

S. no.	Intervention	Method	Duration
1	Interferential therapy	Alternating medium frequency current (4 kHz) amplitude modulated at low frequency, IFT Programme no.12, trapezoid waveform was used	10 min
2	Heat and ice therapy	Local application of moist heat or cold, according to the patient’s sensitivity and symptoms	10 min
3	Ultrasound	Frequency of 1 MHz and intensity of 0.8-1.5 wb/cm^2^ on lower lumbar region	5-6 min
4	Core strengthening exercises	Lower stomach to spines, leg movements, abdominal controlled curls, bridging, wall squats, and single leg bridging	10-15 repetitions (reps), 10-14 reps each side, 10-15 reps, 10-15 reps, 12 reps × 3 sets, 10-12 reps × 2 sets duration: 30 min

**Table 8:** Description of acupuncture points selected for chronic low back pain.

S.no.	Needling point	Location	Depth of needling; method
1	GV 20 (Baihui)	On the vertex of the skull, 5 cun behind the anterior hairline and 7 cun above the posterior hairline in the middle	0.5 cun; oblique needling
2	LI 4 (Hegu)	On the center of a line joining the midpoint of the border of 1st web and junction of the 1st and 2nd metacarpal bones, slightly close to the 2nd metacarpal bone	0.5-1 cun; perpendicular needling
3	UB 11(Dashu)	On the back, 1.5 cun lateral to the tip of the spinous process of the 1st thoracic vertebra	0.5-1 cun; oblique needling
4	GV 3 (Yaoyangguan)	In the center of the interspace between the dorsal spinous processes of the 4th and 5th lumbar vertebrae	0.5-1 cun; oblique needling
5	GV 4 (Mingmen)	In the center of the interspace between the dorsal spines of 2nd and 3rd lumbar vertebrae	0.5-1 cun; oblique needling
6	UB 40 (Weizhong)	Midpoint of the popliteal fossa	1-1.5 cun; perpendicular needling
7	UB 60 (Kunlun)	Central point of the groove between the tendo-Achilles and posterior border of the lateral malleolus of the fibula, at the level of its tip	0.5-1 cun; perpendicular needling
8	St 44 (Neiting)	On the dorsal aspect of the foot 0.5 cun proximal to the web space between the second and third toes	0.2-0.5 cun; perpendicular needling
